# Frequency of Missing TNM Stage Data for Adults With Intellectual or Developmental Disabilities in a Provincial Cancer Registry—A Brief Report

**DOI:** 10.1002/cam4.70579

**Published:** 2025-01-07

**Authors:** Kelly Biggs, Helene Ouellette‐Kuntz, Rebecca Griffiths, Rebecca Hansford, Julie Hallet, Christine Kelly, Kathleen Decker, David E. Dawe, Shahin Shooshtari, Marni Brownell, Donna Turner, Virginie Cobigo, Alyson Mahar

**Affiliations:** ^1^ Department of Public Health Sciences Queen's University Kingston Ontario Canada; ^2^ ICES Ontario Canada; ^3^ Odette Cancer Centre, Sunnybrook Health Sciences Centre Toronto Ontario Canada; ^4^ Department of Community Health Sciences University of Manitoba Winnipeg Manitoba Canada; ^5^ Paul Albrechtsen Research Institute CancerCare Manitoba Winnipeg Manitoba Canada; ^6^ Department of Internal Medicine University of Manitoba Winnipeg Manitoba Canada; ^7^ St Amant Research Centre Winnipeg Manitoba Canada; ^8^ Manitoba Centre for Health Policy University of Manitoba Winnipeg Manitoba Canada; ^9^ School of Psychology University of Ottawa Ottawa Ontario Canada; ^10^ School of Nursing Queen's University Kingston Ontario Canada

**Keywords:** cancer stage, developmental disability, intellectual disability, registry, unknown stage

## Abstract

**Background:**

Adults with intellectual or developmental disability (IDD) are at higher risk for incomplete cancer staging.

**Aim:**

To compare unknown stage data between those with and without IDD.

**Materials and Methods:**

We used the Ontario Cancer Registry linked to administrative health data between 2007 and 2019.

**Results:**

Adults with IDD diagnosed with breast, colorectal, and lung cancer were 1.94 (95% CI 1.52–2.47), 1.90 (95% CI 1.63–2.21), and 2.17 (95% CI 1.86–2.54) times more likely to have unknown cancer stage at diagnosis, relative to those without IDD.

**Discussion:**

The absence of stage data has person‐level and population‐level implications. At the individual level, if stage data are not simply missing from the registry but reflect incomplete or absent diagnostic or staging procedures, this may represent barriers for adults with IDD in receiving curative treatment. At the population level, research using inaccurate or incomplete stage data may lead to unrepresentative health and social system policy decisions.

**Conclusion:**

A better understanding of the cancer diagnostic interval for adults with IDD is needed to develop interventions.

There is a growing body of evidence documenting greater frequencies of missing cancer stage data in cancer registries for persons with severe psychiatric illness [1], older adults [2, 3, 4, 5], those with high levels of comorbidity or complex care needs [3, 6], and adults living in institutionalized settings, such as long‐term care facilities [6, 7, 8, 9]. Cancer staging is a necessary step in the cancer care continuum, directly informing the treatment options available and providing vital information to personalize prognosis and plan for early intervention or palliative care. Cancer stage data are also a critical piece of information for public health surveillance and health services research conducted using cancer registries. Missing stage data in cancer registries are often attributed to limitations in the data collection process. Stage information may be available to the clinical team but unavailable in electronic medical records or pathology reports for a variety of reasons. In these cases, higher rates of missing data are assumed to be the result of errors in data transfer or entry and complete case analyses of individuals with known stage data conducted in research and surveillance. However, stage at diagnosis may also be unknown if the clinical investigations required to formally establish stage are not adequately completed. Both reasons for missing stage information have individual‐ and population‐level implications.

Theoretically, drivers of clinically unknown stage are more likely to result from inequitable systems of care than administrative errors [1]. Adults with intellectual or developmental disability (IDD), lifelong conditions affecting cognitive and adaptive functioning diagnosed at birth or during childhood, may be at higher risk of incomplete or inadequate cancer staging. Plausible pathways to incomplete diagnostic investigation and staging for adults with IDD include communication difficulties, anxiety and fear associated with staging and diagnostic procedures [10, 11], competing health priorities [12, 13], the belief by the provider or support team that staging investigations risks do not outweigh the benefits of treatment [14, 15], the belief that further staging information will not impact the treatment course [14, 15], or the inability to determine appropriate informed consent for the diagnostic procedures [10]. In addition, adults with IDD may be less likely to receive a referral to specialized cancer treatment centers, which may impact the availability of staging data for administrative reasons. Documenting incomplete staging data could direct resources toward clinical interventions and supports at the time of diagnosis. In addition, documenting inequitable stage data availability could support health system policies and procedures aimed at using data from cancer registries and other population‐based resources.

We conducted a population‐based, cross‐sectional study to examine the relationship between IDD status and unknown cancer stage at diagnosis with routinely collected administrative data in Ontario, Canada, linked using uniquely encoded identifiers and analyzed at ICES [16]. Detailed methods and cohort descriptions are available elsewhere [17, 18]. This study received ethical clearance from the Queen's University Faculty of Health Sciences and Affiliated Hospitals Research Ethics Board (#EPID‐691‐19) and approvals from all data holders. Data sources included the Ontario Cancer Registry (OCR), the Registered Persons Database, the Ontario Health Insurance Plan, the National Ambulatory Care Reporting System, the Continuing Care Reporting System, the Home Care Database, the Ontario Mental Health Reporting System, and the Canadian Institute of Health Information Discharge Abstract & Same Day Surgery databases. The study cohort included Ontarians aged ≥ 18 years with breast (female), colorectal or lung cancer recorded in the OCR between January 1, 2007, and December 31, 2019, identified using ICD‐O‐3 codes [19, 20]. The primary outcome was unknown Tumor, Node, Metastasis (TNM) stage at diagnosis. Stage was determined in the OCR through a combination of the provincial Collaborative Staging System and data from individual regional cancer centers, following the American Joint Committee on Cancer and the International Union Against Cancer (AJCC/UICC) TNM stage classification systems. IDD status was determined using lifetime data from hospitalizations, emergency department visits, outpatient physician visits, home care, and continuing care reporting system records [19]. Access to specialized cancer treatment services was measured through registration with one of 14 regional cancer centers in the province. Six major groupings of physical comorbidities were measured and described using the John's Hopkins Aggregate Diagnosis Groups (ADGs) according to the ACG system [21]. Multivariable modified Poisson regression with robust error variance was used to estimate the association between IDD and unknown cancer stage at diagnosis after adjusting for age at diagnosis (continuous), sex (male/female for colorectal and lung cancer cohorts), year of cancer diagnosis, rural/urban, health system planning region, and regional cancer center registration (yes/no). Effect modification analyses by age (group), sex, and regional cancer center, where staging data are more reliably collected and stored in the cancer registry, were performed. Analyses were performed using SAS V9.4 (SAS Institute Inc.).

This study included 104,763 females with breast cancer, 79,448 individuals with colorectal cancer, and 90,344 individuals with lung cancer. Adults with IDD made up 0.4% of the breast cancer cohort, 0.5% of the colorectal cohort, and 0.4% of the lung cancer cohort. Adults with IDD were significantly younger, more likely to live in an urban setting, more likely to live in areas with the lowest income, and less likely to be registered with a regional cancer center (Table 1). The prevalence of unknown stage at diagnosis for adults with IDD was higher than for those without IDD across all cancer sites (breast: 13.1% vs. 7.2%; colorectal: 26.4% vs. 14.1%; and lung: 33.1% vs. 16.2%). Table 2 describes the risk of unknown stage cancer at diagnosis for people with IDD relative to those without IDD. Compared to those without IDD, adults with IDD were almost two times more likely to be diagnosed with unknown stage breast or colorectal cancer and two times more likely to be diagnosed with unknown stage lung cancer. Table S1 summarizes strata‐specific estimates of the effect of IDD on unknown cancer stage across age categories, by sex, and according to whether the person was registered at a regional cancer center. The association between IDD and risk of unknown cancer stage differed by age within breast and colorectal cancers.

**TABLE 1:** Characteristics of adults with and without intellectual or developmental disability (IDD) diagnosed with breast, colorectal, and lung cancer patients in Ontario.

	Breast		*p*	Colorectal		*p*	Lung		*p*
	With IDD	Without IDD		With IDD	Without IDD		With IDD	Without IDD	
	(*n* = 398)	(*n* = 104,365)		(*n* = 428)	(*n* = 79,020)		(*n* = 323)	(*n* = 90,021)	
Mean Age (years, SD)	60.3 (13.9)	61.6 (13.8)	0.05	64.51 (14.59)	68.7 (13.37)	< 0.001	67.1 (12.0)	70.2 (11.0)	< 0.001
Male	0%	0%	—	249 (58.2%)	42,929 (54.3%)	0.11	174 (53.9%)	45,606 (50.7%)	0.25
# of Major comorbidities									
0	88 (22.1%)	42,275 (40.9%)	< 0.001	64 (15.0%)	21,668 (27.4%)	< 0.001	11 (3.4%)	12,557 (13.9%)	< 0.001
1	130 (32.7%)	34,171 (32.7%)		88 (20.6%)	23,074 (29.2%)		47 (14.6%)	24,950 (27.7%)	
2+	180 (45.2%)	27,469 (26.3%)		276 (64.5%)	34,278 (43.4%)		265 (82.0%)	52,514 (58.3%)	
Community income quintile^a^									
Lowest	111 (28.0%)	18,513 (17.8%)	< 0.001	121 (28.3%)	15,874 (20.2%)	< 0.001	100 (31.3%)	22,119 (24.7%)	0.02
2	87 (22.0%)	20,558 (19.7%)		96 (22.5%)	16,445 (20.9%)		76 (23.8%)	20,267 (22.6%)	
3	80 (20.2%)	20,496 (19.7%)		83 (19.4%)	15,749 (20.0%)		53 (16.6%)	17,426 (19.4%)	
4	58 (14.6%)	21,602 (20.8%)		55 (12.9%)	15,438 (19.6%)		56 (17.5%)	15,840 (17.7%)	
Highest	60 (15.2%)	22,934 (22.0%)		72 (16.9%)	15,252 (19.4%)		35 (10.9%)	14,037 (15.7%)	
RIO Score									
Rural	45 (11.3%)	9490 (9.1%)	0.31	73 (17.1%)	9390 (11.9%)	0.001	51 (15.8%)	11,700 (13%)	0.009
Semi‐Urban	190 (47.7%)	50,917 (48.8%)		211 (49.3%)	38,343 (48.5%)		177 (54.8%)	44,579 (49.5%)	
Urban	163 (41.0%)	43,958 (42.1%)		144 (33.6%)	31,279 (39.6%)		95 (29.4%)	33,742 (37.5%)	
Regional Cancer Center Registration	315 (79.1%)	95,892 (92.0%)	< 0.001	183 (42.8%)	47,680 (60.3%)	< 0.001	159 (49.2%)	63,290 (70.3%)	< 0.001

*Note:* Full details on the study cohort available elsewhere [18].

^a^ Missing income data for 264 breast cancer, 263 colorectal, and 335 lung cancer patients, data not presented separately by IDD to suppress small cell sizes, in accordance with privacy regulations.

**TABLE 2:** Main effects table for the association between intellectual or developmental disability (IDD) and unknown stage cancer at diagnosis stratified by cancer type (reference group = without IDD).

	# (%) with unknown stage		RR (95% CI)	ARR^a^ (95% CI)	ARR^b^ (95% CI)
	With IDD	Without IDD			
Breast	52 (13.1)	7532 (7.2)	1.81 (1.40–2.33)	1.94 (1.52–2.47)	1.25 (1.00–1.57)
CRC	113 (26.4)	11,144 (14.1)	1.87 (1.60–2.19)	1.90 (1.63–2.21)	1.40 (1.22–1.62)
Lung	107 (33.1)	14,539 (16.2)	2.05 (1.76–2.40)	2.17 (1.86–2.54)	1.52 (1.32–1.75)

^a^ Adjusted for age (continuous), sex (colorectal and lung cancer), health system planning region [1, 2, 3, 4, 5, 6, 7, 8, 9, 10, 11, 12, 13, 14], rurality (RIO score categories), and year of diagnosis (continuous).

^b^ Additionally adjusted for registration with a regional cancer center (yes/no).

These findings should be considered alongside several limitations. Our unvalidated algorithm to identify IDD relies on health system data and likely misclassified those who are younger and those who use fewer health services. We do not anticipate this would change our study findings. IDD is a heterogeneous umbrella term that encompasses a large, diverse population with various conditions, access to different resources and resulting differences in their levels of disability that has been aggregated into a binary term. Therefore, our findings may differ across IDD subgroups and may not apply to all adults with IDD. Stage data collected in registries for research may not perfectly correlate with clinical information known at the time of diagnosis and treatment decision making., However, missing stage data in cancer registries have been associated with lower odds of receiving systemic therapy in lung cancer, for example [22], suggesting it may be a determinant of quality care.

Our study has documented an increased risk of unknown stage cancer at diagnosis among adults living with IDD. This finding raises important questions regarding equity in cancer care that may begin during the diagnostic evaluation. Our observations are consistent with evidence of lower rates of cancer screening, and more advanced stage cancer at diagnosis for adults with IDD, relative to those without [15, 23, 24]. In our study, the risk of having unknown stage in the registry decreased when registration with a regional cancer center was included in the model but did not differ based on access to specialized cancer services through regional cancer centers. These findings suggest that the barriers to cancer staging are not exclusive to community settings. However, we observed that the youngest and oldest people with IDD and breast cancer and the oldest with colorectal cancer had a significantly higher risk of unknown stage cancer. Further research is needed to understand what mechanisms drive these differences, including the role of comorbid physical health conditions and advance care directives. Importantly, we have also provided evidence that adults with IDD and breast, colorectal, and lung cancer are significantly less likely to be registered at a regional cancer center than those without IDD. This observation necessitates further investigation given the role of specialized cancer centers in delivering high‐quality cancer and supportive services, such as receipt of curative and palliative radiation, access to system navigation support, services from a range of providers including social work and dietetics, as well as entry into clinical trials. This is particularly important considering evidence that adults with unknown stage breast and colorectal cancer living with IDD have a three times greater rate of dying following a cancer diagnosis than those without IDD [17].

Evidence‐based clinical interventions addressing the individual‐level implications of incomplete staging investigations are needed. These may include upstream individual‐level programs for cancer system navigation that bridge social and medical services, such as nurse‐navigation programs initiated within community services before a cancer diagnosis. Or they may include system‐level inclusive strategies and policies that redefine the delivery of person‐centered cancer care to adults with IDD, such as accessible material explaining cancer diagnostic and staging investigations. Our work also identifies farther‐reaching clinical implications for the population‐based evidence base of cancer registry‐based research that contributes to surveillance, treatment guidelines, and local policies, if adults with IDD are excluded due to missing stage data. Future research should investigate elements of the peri‐diagnostic period, including speaking with healthcare and social care professionals to understand why staging data are missing and how the system may be adapted or modified to result in better diagnostic outcomes.

## Author Contributions

**Kelly Biggs:** conceptualization (equal), formal analysis (supporting), methodology (equal), writing – original draft (lead). **Helene Ouellette‐Kuntz:** conceptualization (equal), funding acquisition (equal), methodology (equal), supervision (equal), writing – review and editing (supporting). **Rebecca Griffiths:** formal analysis (lead), writing – review and editing (supporting). **Rebecca Hansford:** methodology (supporting), writing – review and editing (equal). **Julie Hallet:** conceptualization (equal), funding acquisition (equal), writing – review and editing (equal). **Christine Kelly:** conceptualization (supporting), writing – review and editing (equal). **Kathleen Decker:** conceptualization (equal), funding acquisition (equal), methodology (equal), writing – review and editing (equal). **David E. Dawe:** conceptualization (equal), methodology (equal), writing – review and editing (equal). **Shahin Shooshtari:** conceptualization (equal), funding acquisition (equal), writing – review and editing (equal). **Marni Brownell:** conceptualization (equal), funding acquisition (equal), writing – review and editing (equal). **Donna Turner:** conceptualization (equal), funding acquisition (equal), writing – review and editing (equal). **Virginie Cobigo:** conceptualization (equal), funding acquisition (equal), writing – review and editing (equal). **Alyson Mahar:** conceptualization (lead), funding acquisition (lead), methodology (lead), supervision (lead), writing – original draft (supporting), writing – review and editing (equal).

## Ethics Statement

The study received ethical clearance from the Queen's University Faculty of Health Sciences and Affiliated Hospitals Research Ethics Board (#EPID‐691‐19).

## Conflicts of Interest

K.B., H.O.K., R.G., R.H., C.K., K.D., S.S., M.B., D.T., V.C., C.K., and A.L.M. declare no competing interests. J.H. has received payments for speaking honoraria from Ipsen Biopharmaceuticals, Advanced Accelerator Applications, Medtronic, and Brystol‐Myers‐Squibb. D.E.D. has received research grants from AstraZeneca, as well as honoraria for education materials from AstraZeneca, Boehringer‐Ingelheim, and Bristol‐Myers Squibb and participates as an advisory board member for AstraZeneca, Merck Canada, Jazz Pharmaceuticals, Pfizer, Roche, and Novartis.

## Supporting information


**Table S1.** Effect modification of the association between intellectual and developmental disability (IDD) and unknown stage at diagnosis by age, sex, and registration with a cancer center, stratified by cancer type (reference group = without IDD).

## Data Availability

The dataset used in this study is held securely in coded format at ICES. ICES is a prescribed entity under section 45 of Ontario's Personal Health Information Protection Act. Section 45 authorizes ICES to collect personal health information, without consent, for the purpose of analysis of compiling statistical information with respect to the management of, evaluation or monitoring of, the allocation of resources to or planning for all or part of the health system. Legal restrictions and data sharing agreements prohibit ICES from making the dataset publicly available. Access may be granted to those who meet the conditions for confidential access, available at https://www.ices.on.ca/DAS. AM holds an appointment as an ICES Scientist, which enabled access to ICES data. Data access is available to external public sector researchers either through collaboration with an ICES scientist or directly, following project approval, via a secure online desktop infrastructure (see above link for details).

## References

[1] A. L. Mahar , P. Kurdyak , T. P. Hanna , N. G. Coburn , and P. A. Groome , “Cancer Staging in Individuals With a Severe Psychiatric Illness: A Cross‐Sectional Study Using Population‐Based Cancer Registry Data,” BMC Cancer 20, no. 1 (2020): 476. 32460722 10.1186/s12885-020-06943-w PMC7251666

[2] J. Adams , R. A. Audisio , M. White , and D. Forman , “Age‐Related Variations in Progression of Cancer at Diagnosis and Completeness of Cancer Registry Data,” Surgical Oncology 13, no. 4 (2004): 175–179. 15615653 10.1016/j.suronc.2004.08.007

[3] C. M. de Camargo , F. Chapuis , and M. P. Curado , “Abstracting Stage in Population‐Based Cancer Registries: The Example of Oral Cavity and Oropharynx Cancers,” Cancer Epidemiology 34, no. 4 (2010): 501–506. 20452853 10.1016/j.canep.2010.04.012

[4] M. Ramos , P. Franch , M. Zaforteza , J. Artero , and M. Durán , “Completeness of T, N, M and Stage Grouping for all Cancers in the Mallorca Cancer Registry,” BMC Cancer 15, no. 1 (2015): 1–6. 26537005 10.1186/s12885-015-1849-x PMC4632343

[5] J. L. Worthington , S. M. Koroukian , and G. S. Cooper , “Examining the Characteristics of Unstaged Colon and Rectal Cancer Cases,” Cancer Detection and Prevention 32, no. 3 (2008): 251–258. 18804920 10.1016/j.cdp.2008.08.006

[6] S. M. Koroukian , F. Xu , H. Beaird , M. Diaz , P. Murray , and J. H. Rose , “Complexity of Care Needs and Unstaged Cancer in Elders: A Population‐Based Study,” Cancer Detection and Prevention 31, no. 3 (2007): 199–206. 17658225 10.1016/j.cdp.2007.04.002 PMC2577170

[7] C. J. Bradley , J. P. Clement , and C. Lin , “Absence of Cancer Diagnosis and Treatment in Elderly Medicaid‐Insured Nursing Home Residents,” JNCI Journal of the National Cancer Institute 100, no. 1 (2008): 21–31. 18159068 10.1093/jnci/djm271

[8] E. B. Ostenfeld , T. Frøslev , S. Friis , P. Gandrup , M. R. Madsen , and M. Søgaard , “Completeness of Colon and Rectal Cancer Staging in the Danish Cancer Registry, 2004–2009,” Clinical Epidemiology 4, no. sup2 (2012): 33–38. 10.2147/CLEP.S32362 PMC3280862 23115791

[9] R. Yancik , M. N. Wesley , L. A. Ries , R. J. Havlik , B. K. Edwards , and J. W. Yates , “Effect of Age and Comorbidity in Postmenopausal Breast Cancer Patients Aged 55 Years and Older,” Journal of the American Medical Association 285, no. 7 (2001): 885–892. 11180731 10.1001/jama.285.7.885

[10] R. Kiani , A. Vahabzadeh , E. Hepplewhite , et al., “Overcoming Challenges in Diagnosing and Treating Cancers in People With Intellectual Disability: A Case Analysis,” Tizard Learning Disability Review 19, no. 2 (2014): 51–58.

[11] G. Breau , S. Thorne , J. Baumbusch , T. G. Hislop , and A. Kazanjian , “Family Physicians' and Trainees' Experiences Regarding Cancer Screening With Patients With Intellectual Disability: An Interpretive Description Study,” Journal of Intellectual Disabilities 27, no. 1 (2023): 250–265. 35189749 10.1177/17446295211044041 PMC9941798

[12] H. Ouellette‐Kuntz , “Understanding Health Disparities and Inequities Faced by Individuals With Intellectual Disabilities,” Journal of Applied Research in Intellectual Disabilities 18, no. 2 (2005): 113–121.

[13] G. L. Krahn , L. Hammond , and A. Turner , “A Cascade of Disparities: Health and Health Care Access for People With Intellectual Disabilities,” Mental Retardation and Developmental Disabilities Research Reviews 12, no. 1 (2006): 70–82. 16435327 10.1002/mrdd.20098

[14] A. J. Boonman , M. Cuypers , G. L. Leusink , J. Nalldenberg , and H. J. Bloemendal , “Cancer Treatment and Decision Making in Individuals With Intellectual Disabilities: A Scoping Literature Review,” Lancet Oncology 23 (2022): e174–e183. 35358466 10.1016/S1470-2045(21)00694-X

[15] L. Iezzoni , “Cancer Detection, Diagnosis, and Treatment for Adults With Disabilities,” Lancet Oncology 23 (2022): e164–e173. 35358465 10.1016/S1470-2045(22)00018-3

[16] M. J. Schull , M. Azimaee , M. Marra , et al., “ICES: Data, Discovery, Better Health,” International Journal of Population Data Science 4, no. 2 (2020): 1135. 32935037 10.23889/ijpds.v4i2.1135 PMC7477779

[17] R. L. Hansford , H. Ouellette‐Kuntz , R. Griffiths , et al., “Breast (Female), Colorectal, and Lung Cancer Survival in People With Intellectual or Developmental Disabilities: A Population‐Based Retrospective Cohort Study,” Canadian Journal of Public Health 115 (2024): 332–342. 38315327 10.17269/s41997-023-00844-8 PMC11027730

[18] A. L. Mahar , K. Biggs , R. L. Hansford , et al., “Stage IV Breast, Colorectal, and Lung Cancer at Diagnosis in Adults Living With Intellectual or Developmental Disabilities: A Population‐Based Cross‐Sectional Study,” Cancer 130, no. 5 (2024): 740–749. 37902956 10.1002/cncr.35068

[19] E. Lin , R. Balogh , V. Cobigo , H. Ouellette‐Kuntz , A. Wilton , and Y. Lunsky , “Using Administrative Health Data to Identify Individuals With Intellectual and Developmental Disabilities: A Comparison of Algorithms,” Journal of Intellectual Disability Research 57, no. 5 (2013): 462–477. 23116328 10.1111/jir.12002

[20] H. Ouellette‐Kuntz and L. Martin , “Applied Health Research Question Report: Aging Profiles of Adults With and Without Developmental Disabilities in Ontario,” 2014, Toronto, ON: H‐CARDD.

[21] Johns Hopkins University , “The Johns Hopkins ACG® System Version 11.0 Technical Reference Guide,” 2014.

[22] R. Rittberg , K. Decker , P. Lambert , et al., “Impact of Age, Comorbidity, and Polypharmacy on Receipt of Systemic Therapy in Advanced Cancers: A Retrospective Population‐Based Study,” Journal of Geriatric Oncology 15, no. 2 (2024): 101689. 38219331 10.1016/j.jgo.2023.101689

[23] M. Stirling , A. Anderson , H. Ouellette‐Kuntz , et al., “A Scoping Review Documenting Cancer Outcomes and Inequities for Adults Living With Intellectual and/or Developmental Disabilities,” European Journal of Oncology Nursing 54 (2021): 102011. 34517198 10.1016/j.ejon.2021.102011

[24] A. J. Boonman , M. Cuypers , G. L. Leusink , J. Naaldenberg , and H. J. Bloemendal , “Cancer Treatment and Decision Making in Individuals With Intellectual Disabilities: A Scoping Literature Review,” Lancet Oncology 23, no. 4 (2022): e174–e183. 35358466 10.1016/S1470-2045(21)00694-X

