# Supplementary material for: Frequency of Missing TNM Stage Data for Adults With Intellectual or Developmental Disabilities in a Provincial Cancer Registry—A Brief Report
Source: Cancer Med. 2025 Jan 7;14(1):e70579. doi: 10.1002/cam4.70579 (PMC11705482; doi:10.1002/cam4.70579)
Supplement: Supplementary file 1 — Table S1. Effect modification of the association between intellectual and developmental disability (IDD) and unknown stage at diagnosis by age, sex, and registration with a cancer center, stratified by cancer type (reference group = without IDD). [file CAM4-14-e70579-s001.docx]

**Supplementary Content**

***Table S1.*** Effect modification of the association between intellectual or developmental disability (IDD) and unknown stage at diagnosis by age, sex, and registration with a cancer centre, stratified by cancer type (reference group= without IDD)

|  | **Breast** |  | **Colorectal** |  | **Lung** |  |
| --- | --- | --- | --- | --- | --- | --- |
|  | **Adjusted^$^ RR (95% CI)** | **P** | **Adjusted^$^ RR (95% CI)** | **P** | **Adjusted^$^ RR (95% CI)** | **P** |
| **Age at diagnosis** |  |  |  |  |  |  |
| ≤49 years | 2.79 (1.47 -5.26) | 0.02 | 1.41 (0.86 -2.31) | 0.01 | 1.91 (0.87 -4.19) | 0.94 |
| 50-59 years | 0.90 (0.41 -2.00) |  | 1.38 (0.93 -2.03) |  | 2.46 (1.60 -3.78) |  |
| 60-69 years | 1.70 (1.03 -2.78) |  | 1.41 (0.95 -2.10) |  | 2.20 (1.59 -3.04) |  |
| 70-79 years | 1.88 (0.99 -3.59) |  | 2.77 (2.09 -3.68) |  | 2.07 (1.53 -2.80) |  |
| 80 years plus | 3.20 (2.21 -4.64) |  | 2.18 (1.69 -2.81) |  | 1.98 (1.55 -2.53) |  |
| **Sex** |  |  |  |  |  |  |
| Male | -- |  | 1.87 (1.51 - 2.31) | 0.82 | 2.14 (1.71 - 2.68) | 0.83 |
| Female | -- |  | 1.93 (1.56 - 2.40) |  | 2.21 (1.79 - 2.74) |  |
| **Regional cancer centre registration** | | | | | | |
| No Cancer Centre | 1.30 (1.00 -1.67) | 0.78 | 1.45 (1.25 -1.68) | 0.13 | 1.48 (1.28 -1.72) | 0.80 |
| Cancer Centre | 1.21 (0.78 -1.87) |  | 1.03 (0.63 -1.67) |  | 1.57 (1.05 -2.35) |  |

^$^Adjusted for age (continuous), sex (colorectal and lung cancer), health system planning region (1-14), rurality (RIO score categories), and year of diagnosis (continuous)
